# Motivations behind individuals’ energy efficiency investments and daily energy-saving behavior: The case of China

**DOI:** 10.1007/s10368-021-00521-6

**Published:** 2021-10-19

**Authors:** J. K. Perret, V. Udalov, N. Fabisch

**Affiliations:** 1 International School of Management, Im MediaPark 5c, 50670 Cologne, Germany; 2 Bundesnetzagentur, Bonn, Germany; 3 International School of Management, Brooktorkai 22, 20457 Hamburg, Germany

**Keywords:** Environment, Behavior, Energy efficiency, Energy-saving, Environmental motivation, China, Cross-cultural, F64, C26, Z13

## Abstract

The impact of environmental motivations on the individual’s decisions regarding investments in energy efficiency and the adoption of energy-saving habits are analyzed for a representative sample of Chinese inhabitants from the larger Beijing area, replicating a comparative study on Western Europe. For the considered type of energy efficiency investments and daily energy-saving activities similarities and discrepancies between the two regions are discussed in regard to their sociocultural background as well as governmental regulations. The study provides empirical evidence that for Chinese environmental issues if all only play a tertiary role after monetary and regulatory incentives. The findings could suggest that in China policy programs aimed at raising environmental awareness and forming pro-environmental motivations might not lead to an increase in energy efficiency investments and daily energy-saving activities and the Chinese government’s interests in this regard might be better served by implementing corresponding incentives via regulations. In the long-term. However, social peer pressure might affect a change in the Chinese mentality.

## Introduction

The residential sector remains one of the main consumers of energy in almost every country of the world. In 2020 the total final energy consumption in the residential sector corresponded to 6.096.76 bn kWh (EIA 2021) out of a total worldwide energy consumption of 27,238.90 bn kWh. Considering China, the total final energy consumption of the residential sector corresponds to 1,094.90 bn kWh (CEIC 2021) and is after the industrial sector the most energy consuming sector (IEA 2020) having almost doubled during the last decade (EIA 2020). The total Chinese energy consumption in 2020 reached 7,511 bn kWh.

While the residential energy consumption in the period between 1990 and 2012 increased by an average of 11.9% per year (NBS 2015), the most recent data suggests an increase of only 7.1% per year in recent years (NBS 2019).

There are many factors shaping this upward trend in energy consumption, such as an increase in the number of households, greater comfort demanded due to an increase in the standard of living, and an increase in electrical appliances in homes (Eurostat 2013). A deeper analysis of behavior patterns and views on environmental topics impacting this sector are thus of primary importance when discussing issues of climate change.

Households can affect their energy consumption by increasing the energy efficiency of their stock of appliances or by undertaking energy-saving activities. If the aim is to encourage households to reduce energy use, it is important to target determinants of energy use and conservation (Abrahamse and Steg 2009). Motivations that lead households to adopt energy-saving activities like most behavioral patterns are very complex. Economic factors like saving money on energy bills seem to be among the primary incentives influencing environmental friendly investments and daily saving behavior. Nevertheless, environmental motivations and social norms and structures might also play an important role (Allcott 2011; Ek and Söderholm 2010; Frederiks et al. 2015; Niamir et al. 2020; Wolske et al. 2020).

In particular, the environmental motivations behind energy-saving activities might be interesting from a policy perspective (Urban and Ščasný 2012; Wang et al. 2021). Bamberg (2003) considers environmental motivations as situation-invariant orientation patterns, which remain stable independently of whether a particular type of energy-saving provides returns. They can reduce the unintended negative consequences of improved energy efficiency, such as the rebound effect. Furthermore, due to their cross-situational influences, environmental motivation might result in a spill-over of environmentally-friendly behavior to different areas (Whitmarsh 2009).

Considering the relevance of the residential sector in the overall energy consumption mix worldwide, but in particular in China the question can be raised about the relevance of environmental motivations in energy-saving behaviours and environmental friendly investments in China. In this regard this study aims to follow the design of the empirical study by Udalov et al. (2017) detailed in Udalov (2019) that focused on Germany, the Netherlands and Belgium as well as the study by Nie et al. (2019) with its focus on China the methodology adopted follows Vasseur et al. (2019).

The question it strives to answer in this context can be stated as:

What are the factors driving Chinese households’ environmental friendly investments and their energy-saving behaviours?

- The analysis is performed by employing data collected in the scope of the international research project on “Energy Efficiency of Households in Cities”. The implemented data has been collected in China through an online questionnaire. Due to the geographical and also cultural heterogeneity the survey has been conducted solely in the larger Beijing area.
- The next section reviews relevant literature providing a theoretical background on the topic at hand as well as on cultural peculiarities in China. It gives an overview on the state and the general acceptance of environmental awareness in China motivating in detail the conduction of this study. The third section presents the empirical analysis by describing the data set and relevant variables and empirical results. The paper concludes with a discussion of implications for the literature and policymakers.

## Literature review and theoretical background

### Energy-saving behavior

Considering individual energy-saving behavior, a number of studies analyze key determinants. Abrahamse and Steg (2009) and (Abrahamse and Schuitema 2019) divide the key determinants into psychological and sociodemographic factors. Considering the aim of this paper, only those studies which have investigated the impact of environmental motivations on the individual’s decision-making process regarding environmental friendly investments and adoption of energy-saving habits are considered.

Various studies suggest that social factors influence households’ energy-conserving behavior, whereby a social norm is defined as an expectation shared by a group, which specifies behavior that is considered to be appropriate for a given situation (Gardner and Stern 1996; Trotta 2018). Ek and Söderholm (2010) postulate that other people’s attitudes and behavior in electricity saving might influence individuals’ willingness for electricity saving activities. Attitudes develop as a result of cumulative experience and knowledge derived from past exposure to environmental stimuli. More positive attitudes towards energy conservation are associated with higher energy savings (Abrahamse and Steg 2009). Ek and Söderholm (2010) and Zografakis et al. (2010) state that an individual’s attitude towards the environment is an important factor in predicting their electricity saving activities.

Since households’ energy-conserving behavior includes a wide range of activities, it should be noted that the above stated determinants of households’ energy conservation behavior affect different activities in various ways depending on their type and level of their involvement. Jansson et al. (2010) makes a distinction between energy efficiency investments and curtailments that is implemented in later studies like Li et al. (2020) and Niamir et al. (2020) as well.

Energy efficiency investments involve the acquisition of new technologies, low energy appliances or energy efficient systems that need monetary investments. These types of behaviors substitute capital for energy and involve one-time purchase decisions, which are associated with an initial financial expense and a potential for future savings. The latter refers to non-monetary investments that are behavioral changes such as scheduling efforts, turning off lights, cutting down on heating or on air-conditioning and switching off standby mode. Curtailment behaviors are made on an everyday basis and involve frequent efforts, and often result in discomfort for the actor performing the behavior (Jansson et al. 2010). Note that herein the term environmental friendly investments are considered that includes energy efficiency investments but is not limited to them.

Gatersleben et al. (2002) and Whitmarsh (2009) show that actions which are easier to perform (daily energy-saving behavior) are more likely to be driven by psychological factors, while actions which require sacrifice or are connected with considerable monetary costs (environmental friendly investments) are more dependent on conductive circumstances. This idea is discussed as early as 1979 when Kahnemann and Tversky (1979) laid the foundations for the field of behavioral economics where a central assumption holds that long-term investment decisions are governed by rational decision-making while habitual behavior patterns are intuitive in nature and driven by different decision-making processes. However, research that links behavioral economics not only to issues of climate change in general but to individual environmental household behavior is rather scarce with exceptions like Frederiks et al. (2015) or the study by Lehner et al. (2016) who propose nudging as a tool of environmental policy making.

Psychological factors such as values, beliefs and norms, which include environmental motivations, have been identified to be successful in predicting curtailment behavior. Personal norms, experienced as feelings of amoral obligation to act, affect both curtailment activities and low- to medium-involvement purchase decisions. Environmental beliefs in the form of an ascription of responsibility influence curtailment behaviors (Jansson et al. 2010). Eriksson et al. (2006) and Nordlund and Garvill (2003) show that for willingness to curtail personal car use, there is a strong influence of personal norms. Jansson et al. (2010) argue that energy efficiency investments are high-involvement activities because they incur considerable monetary costs and also require time and planning activity for their implementation. These measures rely on external conditions such as economic concern and are less affected by psychological and social factors (Guagnano et al. 1995). Following this line of argumentation, a lower income might be positively related to daily energy-saving activities since these behavior patterns are less financially demanding, even allowing for additional monetary gains.

However, Fischer (2008) shows that curtailment behaviors associated with changing habits and some discomfort on the individual level are not driven by environmental motivations because the environment is not a relevant issue to consider at the time the habit is formed. Urban and Ščasný (2012) indicate that people with higher environmental concern are on average more likely to perform both energy-saving curtailments and environmental friendly investments. Jansson et al. (2010) also delivers empirical evidence that biospheric values (as in considering the effects of one’s own decisions on the biosphere) and personal norms (as feelings of a moral obligation to act) have a strong influence on both high involvement once-off actions and ongoing day-to-day actions to reduce energy consumption.

Since the existing literature does not give a clear answer regarding the impact of environmental motivations, his paper aims to investigate which environmental friendly investments and daily energy-saving activities are affected by environmental motivations.

Considering studies on China with a focus on green purchasing behaviour Zhang and Dong (2020) reviewed 97 papers (2015–2020) „providing empirical research on green purchase behavior “ focussing on individual, social and product related aspects as the three primarily influencing factors. Even though the authors emphasize the „great significance “ of culture to a person’s value system, there is no clear coherence between culture and „green “ purchase behavior. Sheng et al. (2019) enumerate a number of studies that „provided empirical support that cultural values “ influence „consumer behavioral intention when it comes to environmentally friendly products “. They also link traditional Chinese values to „green “ purchase intentions. Overall there is a certain consensus in literature that a collectivistic society tends to more proactively protect the environment as values of being cooperative and caring for others are evaluated higher (Cheung and To 2019).

Regarding a focus on energy-saving behaviours and environmental friendly investments studies are rather sparse with the studies Wang et al. (2011), Ru et al. (2018), Cai et al. (2019), Nie et al. (2019) and Fang et al. (2021) providing some of the few studies concerning Chinese inhabitants. Cai et al. (2019), however, focusses solely on bicycle sharing and Fang et al. (2021) on the existing attitude behaviour gap.

This study thus aims at providing additional insights into this topic and analyses the impact of environmental motivations on individual environmental friendly investments and daily energy-saving behavior in China which before has only been considered by Nie et al. (2019).

### The cultural mindset and environmental thinking in China

China’s economy has grown “by a factor of 30 “ since Deng Xiaoping started the economic reform of the country in 1978. Since then China has become the world’s largest producer of industrial goods (Linster and Yang 2018) and the world’s largest energy consumer. In 2018 China “accounted for 24% of global energy consumption and 34% of global energy consumption growth “ (BP Statistical Review 2019). These economic efforts have had its side effects on China’s environment as industrial expansion was mainly responsible for air pollution, greenhouse emissions and waste (Linster and Yang 2018). Especially the alarming smog situation in China’s major cities has been widely discussed and its influence in health damages has been proven by several studies (Liu et al. 2018). Water and soil pollution are also an issue for serious health concern in China, especially in rural areas (Wu 2020).

These environmental and social challenges induced the Chinese Government to open up to a more sustainable development strategy. As early as the 11^th^ Five-Year Plan the central committee supported a transformation to synchronize economic growth and environmental protection (Zhong and Shi 2020). Especially China’s current 13^th^ Five-Year Plan for Economic and Social Development (2016 – 2020) is explicitly dedicated to the ecosystem and the environment setting goals to „step up ecosystem and environmental protection efforts “ with the overall goal to „help the country grow stronger, and build a Beautiful China “ (Central Committee of the Communist Party of China 2016). As the Chinese government started to actively promote energy saving and environmental consciousness, three questions arise. Since China no longer is a centrally planned economy but transitioned into a market economy the question comes up whether the Chinese economy actively follows the path laid out by the government. However, recently published reports indicate that the “Chinese Communist Party (CCP or the “Party”) is currently strengthening the presence and powers of internal Party organizations located within Chinese companies to ensure greater oversight and influence over China’s commercial sector” to ensure the development of a “modern enterprise system with Chinese characteristics “ (Livingston 2021). President Xi recently again “emphasized the strategic importance of eco-environmental investments for China”. “Environmental regulations and emission standards are becoming stricter”, accompanied with programs, such as “Made in China 2025” (MIC25) to push for rapid advances in domestic innovation” and special “conditions for private companies that engage in energy conservation and environmental protection, e.g., via tax relief (Holzmann and Grünberg 2021). More than 500 environmental non-governmental organizations (ENGOs) and foundations are also working on “raising awareness of green product consumption” (ibid.)

Third, it can be asked whether society is also exerting itself to do so and whether traditional Chinese values are supportive when it comes to environmental protection.

When making assumptions about consumer behavior culture, besides other variables, such as psychological or sociodemographics, it is of „great significance to an individual’s value orientation “ (Zhang and Dong 2020). Cultural values not just structure a person’s lifestyle and have an influence how sustainable issues are evaluated (Sheng et al. 2019), but also influences attitudes which are the cornerstone of behavior. Therefore, a closer look at Chinese values might be helpful for a better understanding of the environmental motivations. Considering the geographical size of China and consequently its cultural heterogeneity, it remains questionable at best if it is possible to extract a single Chinese culture.

Particularly Hofstede’s theories have had the greatest impact on intercultural studies especially when comparing Eastern or Western cultures (Chang et al. 2019), whereas it can be assumed to reflect mostly the industrialized East of China. This argument is strengthened by the results of Xu et al. (2019) even though they primarily focus on the differences between Shangdong and Xinjiang province and by Song and Soopramanien (2019) who consider Beijing as another major city in China that is reflected via the number reported by Hofstede. Huo and Randall (1991) in contrast report significant differences between Beijing and other areas in China and Taiwan; it needs to be mentioned that with a focus on Taiwan, HongKong, Beijing and Wuhan they implement a peculiar focus and the differences between Beijing and Wuhan are the least developed.

The latest model by Hofstede consists of six dimensions: Power-distance-Index (PDI), individualism-collectivism (IDV), masculinity–femininity (MAS), uncertainty avoidance index (UAI), long-term orientation versus short-term normative orientation (LTO) and indulgence versus restraint (IVR) (Hofstede 2011).

The power distance index (PDI) is an indicator for the acceptance of hierarchical relationships and position-related roles. China ranks high in the PDI with a score of 80 which can be interpreted that inequality and hierarchical decision-making by authorities is tolerated (Hofstede and McCrae 2004). A large PDI also implies that subordinates „expect to be told what to do “ and children are taught obedience (Hofstede 2011). Following the Hofstede system China (ICV score 20) is a collectivist culture rather than an individual one. This means that the wellbeing of an in-group or relevant society is of greater importance than the personal goals of an individual. Maintenance of harmony is of central importance, „transgression of norms “ is shame-ridden (Hofstede 2011). China is regarded as an ambitious, success-driven masculine culture (MAS score 66) where material goods are cherished. A relatively low score (30) on the uncertainty- avoidance-index (UAI) indicates a certain pragmatism when laws and rules are considered which can be interpreted as flexibility related to the actual situation. Unknown or unstructured situations are expected and require a higher amount of self-control (Hofstede 2011). China scores high in long-term orientation (score 87) what means that the nation focuses on the future and long-term growth including the ability to adapt traditions to changed conditions. The indulgence versus restraint dimension is defined as* „*the extent to which people try to control their desires and impulses “ (Hofstede Insights 2020). At a low score (24) China is seen as a „restrained “ society which is not primarily focused on leisure time, is regulated by social norms and teaches children to control their impulses (Bluszcz 2016). It also tends to regulate gratification of needs by strict social norms (Hofstede 2011).

In addition to these cultural dimensions which might differ even in a country’s own regions there are some more cultural specifics and traditions in China which go beyond Hofstede’s dimensions. Although China ranks „least religious country, with 7 out of 10 people claiming to be atheists “ (67%) (Gallup International 2017) there still exist the traditional values of its (religious-like) philosophies of Confucianism and Daoism. Especially Confucian values of stability, discipline and social order continues to serve as a „leading source of ideas in China’s policy “ (Zhao 2018) and still influence conceptions of „social harmony “. The popular „Doctrine of the Mean “ emphasizes „harmony with nature and a long term perspective “, which enables people to „achieve/maintain a harmonious co-existence with other individuals and society as a whole “ (Rosker 2013) and is therefore connected to even influence green purchasing intention of Chinese consumers (Sheng et al. 2019). In difference to Confucianism Daoism as China’s oldest philosophy does not focus on social hierarchies, but emphasizes naturalness, simplicity, detachment from desires and „wu wei “, as a principle of not-acting to reach the „Tao “. The latter does not mean passivity, but to follow the natural way of the world without disturbing it. In literature Daoism is even termed a „green religion “ which could promote a sustainable future (Miller 2017).

There is a distinct tendency in traditional Chinese values which can be argued to support a more environmentally benign lifestyle. Caring for the society (collectivism) and a long-term orientation encourage a sustainable lifestyle going easy on resources needed by future generations. Flexibility helps to adopt new environment friendly behaviors and products. A high acceptance of authorities and their political directives by society can support the implementation of the eco-friendly goals of the Chinese 13^th^ Five-Year-Plan. Even a restraint society which teaches their children to suppress spontaneous selfish impulses can help to not immediately buy a new product which is not sustainable or on the other hand even pay more to support environmentally friendly or energy-saving products.

Insofar Chinese cultural values are in terms of content compatible with the sustainable mindset and might positively influence environmental awareness and even green purchasing intentions of Chinese consumers (Sheng et al. 2019). They have been „immersed in a Confucian culture for a long time and pursue social harmony, so they are more likely to implement environmental behavior “ (Zhang and Dong 2020). As the level of economic development of a country increases, a certain value shift can be explored and as security and prosperity increase people tend to behave more individualistic (Beugelsdijk and Welzel 2018).

### Environmental awareness in China

Environmental awareness is highly related to environmental education and „assumed to be an important prerequisite of environmental protection “ (Du et al. 2018).

In the 20-year period between 2000 and 2019 China ranks number one concerning natural disasters (UNDRR/CRED 2020). According to the latest report of the United Nations Office for Disaster Risk Reduction (UNDRR), China „experienced a wide variety of over 500 disaster events including geophysical, hydrological, and meteorological event “ (UNDRR/CRED 2020). In the last decades China was heavily hit by all sorts of disasters which are supposed to be related to climate change such as „typhoons, floods, droughts and sandstorms, storm surges, landslides and debris flows, hailstorms, cold waves, heat waves, pests and rodent disease, forest and grassland fires, and red tides “ (World Bank Document 2020). „Between 1989 and 2018, natural hazards caused the death of 195,820 people, and direct physical losses valued at 11,237 billion Chinese yuan (CNY, in 2018 values), or approximately US$1,698 billion (in 2018 values)“ (ibid).

After the rise of environmental disasters in China the comprehension grew that there obviously is a negative correlation between economic growth and environmental degradation (ibid). Using a survey among individuals from five cities in China, Dai et al. (2014) reveal that the personal experience with extreme weather events increases global climate change beliefs. The „promotion of ecological civilization “ became a strategic political goal and the environmental awareness steadily increased (Broadbent 2018). In China’s capital Beijing over 50% of respondents answered „that they participate in environmental protection activities run by local government and communities “ and almost 70% of Chinese city inhabitants see a direct relation between personal consumption and the environment (Li et al. 2017). Other studies are more conservative and describe the environmental awareness of the Chinese people and their public engagement as low (Du et al. 2018). So far Chinese consumers seem to have at least „a basic recognition of sustainable consumption “, but especially younger urban citizens displayed a strong intention and willingness to support sustainable consumption (Li et al. 2017). In another study comparing East Asian countries, Chinese consumers showed the highest willingness to sacrifice for the environment (Chen and Zheng 2016). A study across ten cities in China reported that over 70 percent (73.3%) of the Chinese consumer would be willing to pay at least a premium under 10% for green products. „The closer the products are related to health and safety “, the bigger is their willingness to purchase slightly more expensive environmentally friendly products (Li et al. 2017). Food is after several scandals unsurprisingly ranking number one, but electric supplies for example might also be bought by almost 39% for 5–10% higher price (ibid.). As the Chinese government heavily promotes new energy vehicles (NEVs) with tax breaks and subsidies, 63% of respondents were interested in buying one (Li et al. 2017).

Fewer people stated „fewer costs “ as a reason to purchase sustainable goods, most people assigned environment conservation, safety and health (Li et al. 2017). As interviewees „measure themselves against a social norm “, their answers „generally contain a component of social desirability “ (Berglund et al. 2020).

There are no longer reservations in China’s government when it comes to declare environmental challenges. On the contrary China has adopted the UN sustainability developments goals and steadily improved its score. It even embedded environmental issues in its political theories as the release of more than 600 papers and books about ecological Marxism shows (Wang et al. 2013). The Communist Party adopted sustainability and endeavors to transform industry and society along their agenda. It established environmental protection bureaus all over the country and empowered sub-national authorities (Linster and Yang 2018). Contrary to these statements China still is the largest emitter of CO_2_ and increased itself emissions by 500 Mt over the last year (IEA 2021) and even though China is forced to cancel plans for foreign coal plants (Reuters 2021) domestic coal power increases by 7% over the last 2 years (IEA 2021). As “air pollution, water scarcity, and soil contamination remain threats to the health and livelihoods of China’s people”, the situation increases dissatisfaction with the government (Maizland 2021). Regime stability however is the most important driver of Party-state leadership and a healthy environment plus economic growth are two central pillars for its success. Therefore “China’s environmental ambitions should be taken seriously. Furthermore; building on past successes (e.g., in wind, solar and e-mobility), China strives to assume global leadership in green technologies and sustainable solutions” (Holzmann and Grünberg 2021). So far it is therefore a predominantly a top-down approach and an inconsistent one at that. Additionally, even in a hierarchic society which highly accepts social norms and authorities there might be more enthusiasm towards environmental protection and co-payment when options were developed to engage in local decision-making processes (Xi 2017). Conservation psychology trying to ascertain what „motivates individuals and communities to act more pro-environmentally “ emphasizes the relevance of stakeholder consultations and participation possibilities in general as they not just foster social learning, but also „improve participants’ individual and collective competencies to engage and tackle environmental challenges “ (Varumo et al. 2020). In this field the role of Environmental NGOs (ENGOs) are quite an important issue to reach people especially on a local level (Badruddin 2015). Due to several factors China already started to open up to environmental NGOs. Organizations like Friends of Nature, Global Village of Beijing or Green Homes are tolerated and could play an increasingly important role to positively influence the subjective norms of consumers from a bottom-up perspective. However, the current political course seems to be holding back greater influence of ENGOs, currently focusing more on raising environmental awareness among young people in school and universities. Particularly problematic issues with no easy solutions are however, left out (Bloomberg 2021). So for ENGOs to fully develop their potential a significant change still lies a long road ahead since even tolerated NGOs are not fully functional. As attitudes significantly influence purchase behavior and also „energy-saving intentions “ (Liu et al. 2020) the opening up for local activities and ENGOs might in the future support the growing awareness towards the environment and its protection (Volpe 2017) So far „the overall strength of ENGO in China is relatively weak and their number, funding, capabilities “ and impact are limited (Xi 2017).

Summarizing, an analysis of the motivations underlying Chinese inhabitants’ behavior with regard to environmental issues, in particular the realization of environmental friendly investments and daily energy-saving behaviors will provide additional valuable insights as China on the one hand aims at developing an environmental mindset among its citizens while on the other hand still being significantly different from Western European countries when the cultural background is considered and results therefore are not apparent. Correspondingly, the present study picks up on the methodology of Udalov et al. (2017) and replicates the study for Chinese participants.

## Empirical analysis

### Methodology

In order to explain the impact of environmental motivations, a probit and an ordered probit specification are applied to the pooled sample of individual responses regarding environmental friendly investments and daily energy-saving activities. The baseline specifications considered are the following:

1 $$\mathrm{Pr}\left({EE\_Investment}_{i}=1\right)=\Phi \left(\alpha \bullet {Env\_Motiv}_{i}+{\beta \bullet Control}_{i}^{^{\prime}}+{u}_{i}\right)$$

2 $$\mathrm{Pr}\left({ES\_Behaviour}_{i}>0\right)=\Phi \left(\alpha \bullet {Env\_Motiv}_{i}+{\beta \bullet Control}_{i}^{^{\prime}}+{u}_{i}\right)$$

Φ(⋅) is the cumulative distribution function of a standard normal distribution. EE_Investment_i_ and ES_Behavior_i_ are the corresponding dependent variables. EE_Investment_i_ ∈ {0, 1} are the binary responses of individual i regarding already completed environmental friendly investments.

ES_Behavior_i_ ∈ {1, 5} are responses of individual i regarding fulfilling daily energy-saving activities. Since EE_Investment_i_ is a dichotomous variable and ES_Behavior_i_ is a polychotomous variable, which is scaled from 1 to 5, we apply a probit and ordered probit model respectively.

Env_Motiv_i_ ∈ {1, 5} is the main independent variable of interest which is a polychotomous variable as well. As the environmental motivation has been measured on a Likert-scale the arguments by Labovitz (1967) and Traylor (1983) hold and it can be treated like an interval-scaled variable and introduced into a regression approach as a single unmodified variable. It needs to be stressed that this study assumes an activitiy-oriented view of environmental motivations. This allows for the possibility that in regard to one activity strong environmental motivations are present whereas in regard to a different one they are almost non-existent.

Control_i_ is a vector of control variables containing socio-economic factors, dwelling-related factors and attitudinal variables and u_i_ are error terms. The coefficient of interest throughout the paper is α, which is the effect of environmental motivations on environmental friendly investment and daily energy-saving behavior.

The study thereby adopts the framework approach and questionnaire construction for studying environmental friendly investments and energy-saving behaviors discussed in depth in Vasseur et al. (2019) and implemented in Udalov et al. (2017); this includes the selection of investments and behaviours as well as all underlying motivations considered herein.

Finally, as long as solely cross-sectional data is implemented the deduction of causal links between investments or behaviors on the one hand and environmental motivations on the other are not possible. However, the results of Udalov et al. (2017) suggest that the direction of effect solely exists as depicted in Eqs. (1) and (2). Equalizing environmental motivations with environmental values in conjunction with (Rokeach 1973) furthermore would imply that in the medium term at least they behave like traits implying again the direction of effects as in Eqs. (1) and (2). Nevertheless, the results as illustrated below should be viewed in light of this cave-at.

### Data source and description of the data set

The data set consists of 548 Chinese citizens from the larger Beijing area that have been interviewed in 2016 via cross-sectional online questionnaires using a slightly modified version of the questionnaire that has been used by Udalov et al. (2017) for their study of three Western European countries.^1^ For the execution of the online survey help by a professional survey agency has been employed. While response validity has to be critically evaluated the use of an online survey and a sample recruited via a professional survey agency assures the best data quality achievable.

Three of the 548 observations are omitted as they report erroneous birth years or at the time of the survey are below 18 years of age. The implemented questionnaire has been professionally translated into Chinese to assure a higher degree of validity. Additionally, slight modifications have been made to the questionnaire before translation. The data set used in the course of this study uses the original version of the questionnaire before translation into Chinese.

While focusing on the larger Beijing area alone leads to a lack of representativeness, this limitation is not seen as very critical for two reasons.

First, as argued in Section 2.3 the Chinese value system can be considered less individualistically heterogeneous than those of Western Europe. Thus, while the survey certainly does not represent the entirety of China, it surely represents the roughly 24 million inhabitants of the larger Beijing metropolitan area. Additionally, it can be assumed that it offers a decent enough approximation of the behavior patterns of the inhabitants of other larger metropolitan areas (i.e. Shanghai, Wuhan etc.).

With an average age of roughly 30 years covering the range from 70 to 20 years even though the distribution is highly leftwards skewed it still partially mirrors the Chinese age distribution with a median age of 34–37 according to NBS (2019) while underrepresenting a bit the age cohort of 30-to-50-year-olds. The share of male participants is roughly 45% which slightly underrepresents males.

The median household net income lies at just slightly below 90,000 ¥ which at the time of the survey would translate into approximately 13,800 $. This puts the participants of the survey in the high-income class when considering China as a whole but only the upper middle-income class when the larger Beijing area is considered. Interpolation of the results from the McKinsey report results in a median net household income of approximately 14,600 $ for urban households alone at the time of the survey (McKinsey and Company 2020). Thus, with regard to the income distribution, at least considering metropolitan areas, representativeness is assured.

Considering that a distinct attitude-behavior gaps persists in the context of surveys regarding environmental aspects as witnessed by studies like Schlegelmilch et al. (1996) and Carrington et al. (2010) and in regard to China in particular in Fang et al. (2021) the phrasing considered in the implemented questionnaire tries to overcome this problem.

Starting with the question “During the past years, what kind of actions were done to improve your house / to save energy:”^2^ which captured the different dimensions of environmental friendly investments the participants give the answers summarized in Table 1.

**Table 1:** Environmental friendly investments

	Yes	No
I House Insulation	84.00%	16.00%
II PV Panels	64.89%	35.11%
III Efficient Boiler	83.10%	16.90%
IV CFLs or LED	91.71%	8.29%
V Efficient Car	63.49%	36.51%
VI Electric Car	60.96%	39.04%
Average Decider Ratio	92.49%	

Regarding all six questions, participants were given the possibility to answer: Yes; No; I don’t know; Not applicable. On average roughly 7.5% did answer that they do not have the possibility to affect changes in their homes. Considering housing insulation, the installation of boilers or the installation of PV panels this is a significant share especially considering that of the 92.5% that are able to affect changes in all cases more than 60% already did so or are planning to. This is considered to be a result first and foremost of the relatively higher disposable income in the Chinese sample and that 70.28% own the house they live in.^3^ Second, due to cultural differences house and car ownership is viewed differently in China than for example in Western Europe. A cultural bias exists in China where the ownership of a car, especially an electric car and one’s own apartment is seen as a required status symbol in particular regarding younger males. The high shares, as in the case of the demand for electric cars, become obvious considering the policy of the Chinese government regarding new car registrations. Nevertheless, a share of roughly 61% does not really represent the Beijing average where estimates for the share of electric vehicles range between 6 to 8% (Jin and He 2019). Assuming that participants rightfully answered the questionnaire the upwards bias seems to be mainly a result of the sample’s focus on the upper stratum of Bejing’s society as already hinted at above.

With regard to investments concerning one’s own home the Chinese seems to be considerably advanced. This argument however disregards potential prior situations. While efficient energy-saving insulation in generally more developed countries has been common in the last decades and has continuously been advocated for this trend only started in China. Thus, China started from a different level and continued change or upgrading supposedly has been easier for China. To boost this change the Chinese government issued a number of regulations regarding requirements for house insulation or a ban on incandescent light bulbs, finalized in the period the survey has been conducted (LED Inside 2016).

Opposed to environmental friendly investments which usually represent significant long-term investments energy-saving behavior is short-term oriented and not accompanied by significant monetary investments. In the survey energy-saving behavior has been measured via six items each represented by a five-point scale^4^ – resulting in a theoretical mean of 3. Each of the item starts with the question “How often do you…”. The six items have been answered by 83.03% of all participants. Table 2 summarizes the average scores.

**Table 2:** Daily energy-saving behaviors

	Averaged Score
I Turn heat down at night	2.62
II Close windows while heating	1.92
III Turning lights when leaving	1.61
IV No appliances on stand-by	1.95
V Cycle short distances	2.05
VI Use public transport/car pool	2.13

All items scored significantly below the theoretical mean of 3 (p-values below 0.001). Combining the results from the two tables it seems that Chinese people are more open to enact significant investments than adjust their daily behavior patterns when considering sustainability options.

One explanation for this can be the housing and car or transport situation in China. Alternatively, while energy-saving behavior is mainly driven by environmental intentions the monetary factor aside from other non-environmental aspects is a relevant factor when facing long-term investments as has been argued in Section 2.1 and stressed by Udalov et al. (2017). At this point it still remains an open question whether this view is really shared by the participants or whether their intentions are perhaps shaped by external drivers.

### Results

In this part two sets of regressions are estimated to answer the main research objective of this study, in how far environmental motivations drive energy-saving investments and behaviors. First the six investment types are considered and in the second set the six energy-saving behavior patterns.

For the different types of investment the dependent variables resulted from the questions: “During the past years / months…” (P5Q) and the primary independent variables resulted from the questions: “How often do you…” (P8Q). Controls that have been used were, aside from socio-economic aspects like gender, age (via birth year), income and education, whether housing is owned and how well it is isolated.

Finally, the questionnaire contained a set of 28 questions about personal views on economical and ecological development. Those questions pick up a number of aspects introduced in Section 2.3 when environment awareness in China has been discussed. They thus offer a decent measure for the presence of environmental awareness on an individual level. 27 of the 28 questions were used as inputs in a factor analysis^5^ (KMO value of 0.902) to extract five factors; one question had to be left out due to a low communality value. The second extracted factor can be interpreted as a construct measuring an “Active interest in environmental issues” and the fourth extracted factor can be interpreted as a construct measuring “Worries about climate change and the future”. The fifth factor combines experiences with “Extreme weather events and pollution”. These three factors – summarizing the most critical aspects in regard to environmental awareness—were implemented as additional variables (omitting the remaining two) since climate change in China reveals itself mainly through diverse extreme weather events, but pollution is a common occurrence in particular in the Beijing area. Using these controls allows to be as close to the analysis of Udalov et al. (2017) as possible having to accept only slight changes in the implemented controls. Some variables implemented by Udalov et al. (2017) were available in the Chinese data set as well but had to be omitted due to problems with multicollinearity.

Referring to the model from Section 3.1 the main variables of interest remain the P8Q variables. Therefore, Table 3 summarizes the marginal effects of the regression results from probit regressions for the P8Q variables (for the categories: yes and no), including standard errors in parentheses, in the first row. In the second row the R^2^ values for the regression are reported. Asterisks report the significance level of the corresponding coefficients (t-tests) or the overall model quality (χ^2^-tests): No asterisk > 10%; * < 10%; ** < 5%; *** < 1% (Tables 3, 4, 5, 6, 7).

**Table 3:** Probit regression – environmental motivation on environmental friendly investments

Investment	Marginal Effects
I House Insulation	-0.044** (0.021) 0.077*
II PV Panels	-0.019 (0.024) 0.112***
III Efficient Boiler	-0.030 (0.019) 0.063
IV CFLs or LED	-0.018 (0.011) 0.103
V Efficient Car	-0.044* (0.025) 0.139***
VI Electric Car	-0.051** (0.026) 0.093***

It can be noted in advance that all R^2^ values are very low which was to be expected since investments and behaviour patterns can be considered rather noisy. Considering the t-tests the table indicates that only with house insulation, more efficient and electric cars environmental motivations have a significant impact on the decision to realize an energy efficient investment. For PV panels the χ^2^-test reports a significant explanatory power of the model but the environmental motivation still do not play any role.

More interesting however is the consistent negative sign in all six cases. Translated this signifies that the particular option is only realized if there exists a lower environmental inclination in this regard. At first glance these results seem highly counter-intuitive. Looking at them from a different angle, interpreting a negative sign for environmentally oriented intentions as a positive indicator for more monetary or otherwise oriented incentives makes the results more understandable. While house insulation and a more efficient car offer distinct monetary incentives for their realization, getting an electric car, in particular in the Beijing area, is one of the few ways to get a new license plate. The relevance of the license plate police of the Chinese government as well as additional regulations of the usage of cars in critical air conditions in determining the motivation of Chinese consumers to buy an electric or more efficient car can also be evidenced empirically. Participants were questioned in how far the licensing policy, subsidies and driving restrictions impacted their purchase of a “new-energy car”. All of these items are strongly (p-values = 0.000) related to the decision to buy an electric or a more energy-efficient car insofar that participants who bought such a car primarily agreed to the three aspects as a main driver of their purchase.

Regarding house insulation and CFLs or LED bulbs the role of government regulations has already been stressed in the previous chapter and considering the results for the purchase of electric and energy-efficient cars it is likely that comparable reasons dominate here as well even though the participants were not questioned accordingly.

Controlling for age and marital status (as via the question “Do you have a partner.”) does not impact the reported results significantly. In particular having a partner only has a significantly positive impact on the decision to insulate the house and a weakly significant impact on installing a more efficient boiler. Regarding age (via the birth year) there is a weakly significant effect on the installation of PV panels and the purchase of more efficient cars. In both cases the effect is positive implying that younger people are more open towards realizing these investments.

To show that environmental issues are not totally irrelevant in the decision-making process, although in a more indirect way, the marginal effects of the construct “Active interest in environmental issues” are summarized in Table 4. In this regard this study picks up on a result by Yushkova and Feng (2017) who show that a pro-environmental attitude per se can act as a motivation in enacting pro-environmental behaviors, in particular in a sample of young Chinese participants.

**Table 4:** Probit regression – interest in environmental issues on environmental friendly investments

Investment	Marginal Effects
I House Insulation	0.052** (0.023)
II PV Panels	0.115*** (0.033)
III Efficient Boiler	0.038* (0.023)
IV CFLs or LED	-0.000 (0.015)
V Efficient Car	0.102*** (0.032)
VI Electric Car	0.087*** (0.033)

These results, except for CFLs or LEDs are significantly positive. Combining these results with the ones from Table 3. the picture emerges that while environmental issues are not the main drivers for Chinese participants in realizing energy efficient investments, people more actively interested in environmental issues are also more open to realizing energy efficient and thus environmentally friendly investments. The exception of CFLs or LED bulbs can be explained by the time the survey has been executed which falls into the final phase of the phase out of using incandescent light bulbs.

Switching from energy efficient investments to energy-saving behaviors Table 5 summarizes the results of ordered probit regressions for the questions “How often do you…” (P6Q) as dependents and the corresponding environmental motivation variable (P8Q) as the main independents. The pool of control variables remains the same.

**Table 5:** Ordered probit regression—environmental motivation on energy-saving behaviors

Behavior	Marginal Effects
I Turn heat down at night	0.026* (0.015) 0.013
II Close windows while heating	0.001 (0.021) 0.016
III Turning lights when leaving	0.003 (0.020) 0.014
IV No appliances on stand-by	0.001 (0.020) 0.014
V Cycle short distances	0.013 (0.020) 0.013
VI Use public transport/car pool	0.022 (0.018) 0.023*

With the sole exception of “Turn down the heat at night” no other behavior is impacted significantly by participants environmental motivations and even in this case the overall model cannot be considered to have any explanatory power at all.

Controlling for age and marital status only leads to a significant impact of age on the use of public transport with older people being more open to it. In general considering the two variables does not change the reported results significantly.

As argued earlier behavior patterns might be shaped by external drivers as well. One of these external drivers can be found in the housing situation per se. In the South of China heating as such is no particular problem and energy-saving behaviors primarily concern the use of air conditioning. In the larger Beijing area as in most Northern areas of China heating, though an important aspect of housing incurs only a prepaid lump-sum payment and behaviors might reflect on this. This worsened as there is almost no competition on the Chinese energy market making it nigh impossible for Chinese consumers to look for more suitable and more sustainable energy and thus as well heating providers. It can furthermore be mentioned that 38.7% of the participants state that their primary source of heating is via central heating. This share is higher for younger than for older participants. Still 78.4% of the participants state that they can switch off heating if they desire to with an additional 6.6% not knowing it.

In a similar line of argumentation the fifth and sixth point might strongly be related to the public transport infrastructure available and usable as well as the personal availability of alternative means of transportation like owning their own car, potentially and electric one that would offer them additional privileges. This argument is supported by a negative correlation between the investment in an electric car and the use of public transport. However, this correlation is very weak and highly insignificant.

Considering the results from the environmental friendly investments the interest in environmental issues is considered as well and the corresponding marginal effects are reported in Table 6.

**Table 6:** Ordered probit regression – interest in environmental issues on energy-saving behaviors

Behavior	Marginal Effects
I Turn heat down at night	0.014 (0.018)
II Close windows while heating	-0.024 (0.026)
III Turning lights when leaving	-0.010 (0.029)
IV No appliances on stand-by	0.016 (0,027)
V Cycle short distances	0.019 (0.025)
VI Use public transport/car pool	0.002 (0.022)

Again, all coefficients are highly insignificant. Thus, a more pronounced interest in environmental issues does not translate into more environmental friendly behaviour. Combining these results with the fact that the coefficient of determination never goes beyond 1.6% shows that energy saving behaviors in China cannot be suitably described by environmental motivations or interests in environmental issues. While the coefficient of determination in the context of probit estimation can be critically viewed the very low R^2^ values show that even considering the controls that were implemented the behavior pattern of the Chinese participants remain very hard to grasp. Referring back to the discussion of the results summarized in Table 2 this however comes as no surprise as they already show that energy-saving behavior per se is unimportant; all behaviors ranked on average significantly below the average rating of 3. In this regard the results would oppose those of Yushkova and Feng (2017) that a pro-environmental attitude affects pro-environmental behaviors. Harris (2006) in his overview on publications considering the question shows that this is not unanimous.

Summarizing, energy-saving behavior in China is not very common and in those cases that is occurs it is not driven by an environmental mindset. Thus, little has changed since the study by Chan (1999) about two decades earlier. This result validates a deeper look into the motivations underlying Chinese energy-saving behaviors. In this regard an additional set of ordered probit regressions has been conducted using as dependent variables a set of eight items that measure the importance of other motivations. In Table 7 for each of the six behaviors B I to B VI a regression is estimated with the table listing the marginal effects and the corresponding standard errors as well as the coefficient of determination for each of the six models.

**Table 7:** Ordered probit regression—motivation for energy-saving behavior

	B I	B II	B III	B IV	B V	B VI
Saves money	0.131*** (0.041)	0.200*** (0.050)	0.252*** (0.054)	0.246*** (0.049)	0.157*** (0.048)	0.144*** (0.043)
Reduce global warming	0.127*** (0.039)	0.192*** (0.050)	0.196*** (0.052)	0.114** (0.053)	0.243*** (0.046)	0.180*** (0.043)
Reduce energy consumption	-0.008 (0.046)	0.206*** (0.060)	0.264*** (0.060)	0.092 (0.062)	-0.043 (0.054)	0.107** (0.045)
Someone asked me to	-0.157*** (0.042)	-0.068 (0.076)	0.041 (0.064)	-0.155** (0.071)	-0.166** (0.070)	-0.129** (0.057)
Moral thing to do	0.065 (0.053)	0.164*** (0.056)	0.141*** (0.051)	0.047 (0.060)	0.043 (0.061)	0.068 (0.060)
People I know do it	-0.112** (0.047)	-0.066 (0.070)	-0.173** (0.077)	-0.179*** (0.065)	-0.009 (0.081)	-0.074 (0.067)
Feel good doing it	-0.068 (0.056)	0.112 (0.071)	0.185*** (0.061)	0.008 (0.090)	0.018 (0.064)	0.147* (0.079)
Other people approve	-0.058 (0.100)	0.015 (0.173)	0.024 (0.121)	-0.243** (0.114)	-0.055 (0.101)	0.107 (0.140)
R^2^	0.084***	0.090***	0.138***	0.109***	0.061***	0.076***

The table shows that while environmental issues like “Reduce Global Warming” still play an important role, only with regard to cycling and public transport do they have the strongest single effect. Aside from this factor the monetary aspect – directly via “Saves money” and indirectly via “Reduce energy consumption”—is of consistently significant importance which coincides with the results of Li et al. (2017) and the arguments given in Section 2.3. One aspect that has not been considered before but when referring to Section 2.2 becomes obvious, is the social aspect – directly evidenced via “Someone asked me to”, “People I know do it” and “Other people approve” and indirectly as well via “Moral thing to do”. As the respective signs however are consistently negative it is more prudent to talk about unsocial motivations as drivers.

It can thus be concluded that energy-saving behaviors in China are mainly driven by social behaviors and norms in addition to expected monetary gains. This effect has been discussed in detail in Section 2.3 and is also evidenced by Yushkova and Feng (2017) and determined by Li et al. (2017) as one of the two main drivers of environmentally conscious behaviour. Environmental issues though not fully irrelevant only play a tertiary role.

### Chinese behavior patterns vs. western behavior patterns

Due to differences in the questionnaires, the approach implement in this study does not perfectly match the one by Udalov et al. (2017) as different sets of control variables had to be used and the main independent variables used different scales as well.

Despite methodological differences, both studies ask the same questions and results can be compared at least on a general level.

While in Table 3. the marginal effects are negative (although mostly insignificant) Udalov et al. (2017) report highly significant and positive impacts for Western Europe. As this table considered environmental friendly investments it can be concluded that environmental motivations play an important role in Western Europe while in China the question cannot be answered as easily. While the environmental motivation is no direct driver behind energy efficient investments it has additionally been argued that more environmentally interested and concerned participants also more often realize these investments.

Referring back to chapter 2 these results make sense considering that due to regulations impacting in particular inhabitants of the larger Beijing area the purchase of a new car, more efficient or electric, is driven mainly by government subsidies and the chance to acquire a license plate faster than via the ordinary channels. These extrinsic motivations, although subsidies for electric cars exist, are not that prevalent in Western European countries in general.

Regarding housing insulation it is mainly government regulations that drive the investments into better insulated house as compared to environmental motivations (Yu et al. 2014). Similar arguments can be raised with regard to the installation of CFLs or LED bulbs. The time of the survey falls into the fourth phase of the country’s incandescent bulb phase out, 2014 to 2016. After completion of this final phase all types of incandescent bulbs have to be replaced by CFLs or LED bulbs (LED Inside 2016).

In all four cases the main driver behind the investment supposedly has been either a regulatory issue or a potential monetary or time advantage. Considering the high power distance scores for China, evidenced via a strong belief in the central government (Section 2.4), especially the relevance of regulatory issues makes sense; even more so since a low uncertainty avoidance score, as stated in Section 2.2, hints at a population that might be looking to the government for a regulatory framework. That this however does not preclude environmentally oriented people to be more open for investments is evidenced by the results from Table 4.

Considering the strong social norms in China, related in Section 2.2 to the low indulgence and high restraint score, the negative signs of the factors “Someone asked me to” and “People I know do it” can at least in part be explained.

Turning to energy-saving behavior the results from Table 5 show that the relevance of environmental motivations is almost negligible in China as compared to the Western European countries. This result is strengthened by the results from Table 6 that show that even more environmentally interested participants are not interested in realizing more energy-saving behavior patterns. As such the results contradict the findings for Western countries as detailled in Section 2.1 (Jansson et al. 2010) and as well those for China where Fan et al. (2019) show that in Shanghai environmental motivations foster the sorting of waste. Since the sorting of waste is different from the behaviours considered in the course of this study and as argued in Section 2.2 there are cultural difference between the different Chinese regions (Huo and Randall 1991) the results at least valid a more in-depth study on the topic.

It has already been argued that one of the main problems regarding heating can be found in the presence of central heating in the Northern areas of China^6^ as well as prepaid lump-sum payments for heating that remove as well any kind of monetary incentive to more responsible heating behaviors. A positive outlook in this regard can be found in the Hebei Clean Heating Project that is financed by a $ 100 million loan from the WorldBank. It only remains to be seen if the money is used efficiently and accordingly achieving its original goal.

As argued in Section 2.3, ENGOs in China work in fostering an environmental mindset and get people to change their behavior patterns. However, at the moment many of them still only play a marginal role in the Chinese society (Wen 2014). Referring to the results in Table 7 it is primarily monetary incentives and social norms and established behavior patterns that shape ecologically helpful behavior. Thus, a potential point for ENGOs to take hold in might include behavior changes via the establishment of more environment-friendly social norms.

The results integrate well into the picture painted in Section 2.3 that on the one hand in China an environmentally oriented mindset is not yet very developed. On the other hand, similar to environmental investments it is the social and organizational environment that forms behavior patterns.

The importance of the social norms for the adoption of energy-saving behaviors can be understood via the high scores in collectivism that China as compared to Western European countries reaches. While the relevance of social norms in the context of pro-environmental behaviour with links to the general role of social norms as considered in the context of new institutional economics theory (Menard et al. 2005) is discussed broadly in the literature (Czajkowski et al. 2014) a distinct focus on China and the Chinese population still remains underresearched.

Considering what the Chinese government can do to enact a pro-environmental behavior in their population appealing to the social and collectivistic mindset would be an indirect and long-term oriented strategy. As can be seen from the investments a system of regulations and benefits seems to be best suited to address these issues. As monetary motivations are at the forefront of the Chinese customers decision-making processes and since approximately 77.8% of all respondents that answered the corresponding question are in favor of for example recycling fees on products the introduction of a corresponding tax on environmentally harmful products or on unsustainable behavior in general might be a possible way to target these behaviors.

Summarizing there still remains a significant amount of work to be done in China by ENGOs as well as by the central government before the Chinese mindset with regard to environmental issues is as developed as the Western European ones and might have an impact on behavior patterns or long-term investments. However, as evidenced in particular by the results for the environmental friendly investments but as well by the relevance of the aspect “Reduce global warming” as a motivation for more energy-saving behaviors the first steps are already taken and first considerations have taken hold in the Chinese public, at least in the larger Beijing area. This line of thought coincides with the general idea behind the environmental Kuznets theory (Dinda 2004). Since the focus on a greener growth strategy and corresponding regulations have only recently been introduced by the Chinese government and the general population has not yet had the chance to properly internalize these strategies. In comparison in Western Europe the sustainability idea has already been considered for some decades. In does therefore not surprise that environmental-friendly behaviour in Chinese is less intrinsically motivated and more extrinsically enforced.

## Conclusions

### Key insights and applications

Considering the results as summarized in Section 3.4 leads to the insight that environmental policy as conceived in Western European building on raising environmental awareness and thus psychological factors as the environmental motivation for realizing more energy efficient investments or adopting energy-saving behaviors will not be as effective in a Chinese context.

For the Chinese government this does not mean that it is without options when it aims at reducing its population’s environmental footprint. As has been argued in Sections 3.3 and 3.4 at the moment government policy in the form of subsidies, advantages or regulations already works. Thus, at the moment it is a more direct approach to fixing environmental issues that promises to be more effective for China as compared to the indirect approach via the population’s inherent environmental motivation as is the case in Western Europe.

Regarding environmental motivations as such, if the Chinese government should aim at raising awareness and foster behavior changes it can build on the social norms inherent in its culture as in a strongly developed collectivism. A more direct procedure to raise environmentally beneficial behaviors would be by linking them to the social credit system (SCS) which had been at least in part been planned to be realized by 2020, before being disrupted by the COVID-19 pandemic Interestingly, a recent study based on an online survey of 2209 Chinese people found a surprisingly high degree of approval of SCSs across respondent groups with the highest rates found among wealthier, better-educated and urban residents (Kostka 2019). With regard to the criticism of the social credit system and its implementation, applying it to environmental issues needs to assure beforehand that it will not further disadvantage some members of society, in particular via the introduction of unreasonable expectations towards the correct environment friendly behaviour.

Considering that energy-saving behaviors are also shaped by external factors the Chinese government might reflect on the payment structure for heating services and switch from prepaid lump-sum payments and central heating to individually regulated heating on a pay-for-use basis.

### Limitations and outlook

While the study can be considered to build on a stable statistical background and a partially representative sample. The sample still is the most limiting factor of this study. While a sample size of 545 participants can be considered good enough, even if reduced in the course of the regression analysis to roughly 300, it still bears the problem that all participants stem from the larger Beijing area. In a similar line of argumentation in Section 3.2 it has been stated that income and education-wise the sample represents the upper-middle or lower–upper class. As such it is very hard to draw conclusions for the whole of China.

Furthermore, the analysis in this study could only incorporate cross-sectional data and even though arguments in favor of the underlying causal structure have been given a long-term panel study might pick up on additional effects and would allow to explicitly test the assumption of causal effects in the model.

Along a similar argument implementing choice or willingness-to-pay experiments would not only offer the chance to establish causal effects but would furthermore allow for deeper insights into the participants underlying motivations.

As seen in the analysis, environmental issues, experiences or a general environmentally oriented motivation are very weak predictors of energy-saving investments or behaviors. If this is already the case for the upper-middle class the question has to be answered in how far this might become an even more severe problem for the lower classes. At this point there certainly remains a large potential for additional research. Which will not only have to concern itself with analyzing the presence of environmental, energy-saving motivations and their effect on behavior but as well with the question of how the Chinese government might impact behavioral patterns of these classes to adopt more beneficial behaviors.

When considering the motivations underlying daily energy-saving behaviors the results of this study point to potentially unsocial motivations being one of the reasons behind the adoption of certain behaviors. While this results conflicts with the discussion of Chinese culture as introduced in Section 2.2 it may point to other behavior patterns prevalent in Chinese society not covered by standard approaches to cultural indicators like Hofstede’s five dimensions. To get a better understanding of this aspect a more in-depth study is regarding which however is beyond the scope of this paper.

## Footnotes

1 The questionnaire construction as well as the collection of the survey data have been realized within the scope of the project “Energy Efficiency of Households in Cities: A Multi-method Analysis” led by Maastricht University and the Chinese Academy of Sciences.

2 The phrasing of this question assured that participants are asked about already enacted investments and not planned ones. This assures that actual behaviors and not just attitudes are captured.

3 It surprises a bit that only 31.4% of the participants state that they have a separate boiler.

4 Again the phrasing has been designed to reflect on actual behaviors instead of on attitudes about the topics at hand.

5 The use of factor analysis allowed for the extraction of factors—latent variables—from a set of items that represent distinct underlying concepts. A more detailed introduction into factor analysis can be found in Hatcher (2013).

6 Still, considering that 78.4% of the participants state that they can switch off heating relativize central heating as a major source against a sustainable heating behavior.
